# The effects of airway pressure release ventilation on respiratory mechanics in extrapulmonary lung injury

**DOI:** 10.1186/s40635-015-0071-0

**Published:** 2015-12-22

**Authors:** Michaela Kollisch-Singule, Bryanna Emr, Sumeet V. Jain, Penny Andrews, Joshua Satalin, Jiao Liu, Elizabeth Porcellio, Van Kenyon, Guirong Wang, William Marx, Louis A. Gatto, Gary F. Nieman, Nader M. Habashi

**Affiliations:** Department of Surgery, SUNY Upstate Medical University, 750 E. Adams Street, Syracuse, NY 13210 USA; Department of Trauma Critical Care Medicine, R Adams Cowley Shock Trauma Center, University of Maryland School of Medicine, Baltimore, MD USA; Department of Biological Sciences, SUNY Cortland, Cortland, NY USA

**Keywords:** Airway pressure release ventilation (APRV), Low tidal volume ventilation, Lung injury, Chest wall elastance, Transpulmonary pressure

## Abstract

**Background:**

Lung injury is often studied without consideration for pathologic changes in the chest wall. In order to reduce the incidence of lung injury using preemptive mechanical ventilation, it is important to recognize the influence of altered chest wall mechanics on disease pathogenesis. In this study, we hypothesize that airway pressure release ventilation (APRV) may be able to reduce the chest wall elastance associated with an extrapulmonary lung injury model as compared with low tidal volume (LVt) ventilation.

**Methods:**

Female Yorkshire pigs were anesthetized and instrumented. Fecal peritonitis was established, and the superior mesenteric artery was clamped for 30 min to induce an ischemia/reperfusion injury. Immediately following injury, pigs were randomized into (1) LVt (*n* = 3), positive end-expiratory pressure (PEEP) 5 cmH_2_O, *V*_t_ 6 cc kg^−1^, F_i_O_2_ 21 %, and guided by the ARDSnet protocol or (2) APRV (*n* = 3), *P*_High_ 16–22 cmH_2_O, *P*_Low_ 0 cmH_2_O, *T*_High_ 4.5 s, *T*_Low_ set to terminate the peak expiratory flow at 75 %, and F_i_O_2_ 21 %. Pigs were monitored continuously for 48 h. Lung samples and bronchoalveolar lavage fluid were collected at necropsy.

**Results:**

LVt resulted in mild acute respiratory distress syndrome (ARDS) (P_a_O_2_/F_i_O_2_ = 226.2 ± 17.1 mmHg) whereas APRV prevented ARDS (P_a_O_2_/F_i_O_2_ = 465.7 ± 66.5 mmHg; *p* < 0.05). LVt had a reduced surfactant protein A concentration and increased histologic injury as compared with APRV. The plateau pressure in APRV (34.3 ± 0.9 cmH_2_O) was significantly greater than LVt (22.2 ± 2.0 cmH_2_O; *p* < 0.05) yet transpulmonary pressure between groups was similar (*p* > 0.05). This was because the pleural pressure was significantly lower in LVt (7.6 ± 0.5 cmH_2_O) as compared with APRV (17.4 ± 3.5 cmH_2_O; *p* < 0.05). Finally, the elastance of the lung, chest wall, and respiratory system were all significantly greater in LVt as compared with APRV (all *p* < 0.05).

**Conclusions:**

APRV preserved surfactant and lung architecture and maintenance of oxygenation. Despite the greater plateau pressure and tidal volumes in the APRV group, the transpulmonary pressure was similar to that of LVt. Thus, the majority of the plateau pressure in the APRV group was distributed as pleural pressure in this extrapulmonary lung injury model. APRV maintained a normal lung elastance and an open, homogeneously ventilated lung without increasing lung stress.

## Background

Acute respiratory distress syndrome (ARDS) is a syndrome encompassing a broad range of phenotypes yet is often studied clinically as a single disease phenotype. ARDS can be broadly sub-classified into pulmonary versus extrapulmonary ARDS, the ultimate pathology of which may be similar; however, the etiology and physiology of the two subtypes are distinct [1]. Despite these physiologic differences, many of the randomized controlled trials evaluating the impact of ventilator strategies on the incidence and mortality of ARDS analyze patients with pulmonary and extrapulmonary ARDS combined, usually with a preponderance of pulmonary ARDS [2–8], and despite decades of study, the mortality associated with ARDS has not changed since 1994 [8]. Therefore, in order to establish strategies to improve ARDS incidence and mortality, it may be necessary to stratify patients according to ARDS physiology rather than generalizing ARDS as a single phenotype.

Patients with extrapulmonary ARDS are at particular risk for alterations in chest wall mechanics, whereas patients with pulmonary ARDS are less likely to have increases in chest wall elastance (*E*_cw_), with the majority of the pathology associated with increased lung elastance (*E*_l_). The mechanism of increased chest wall elastance in extrapulmonary ARDS is a combination of chest wall edema and increased intra-abdominal pressure (IAP). In patients with normal *E*_cw_, airway opening pressure closely resembles the lung-distending (transpulmonary) pressure, but in patients with an increase in *E*_cw_, a greater portion of the airway opening pressure is generated as pleural pressure, leading to a lower transpulmonary pressure [9]. Chest wall elastance represents only a small fraction of the respiratory system elastance (*E*_rs_) in patients with ARDS with normal chest wall elastance [9]; however, in patients with altered chest wall mechanics, the *E*_cw_ to *E*_rs_ ratio ranges from 20 to 80 % [10]. This great variability demonstrates the fallibility of targeting airway opening pressures without taking the *E*_cw_ and transpulmonary pressure into consideration.

In previous animal studies of extrapulmonary ARDS, airway pressure release ventilation (APRV) was associated with higher tidal volumes and plateau pressures [11, 12], which has raised concern for APRV potentially placing undue stress on the lung. In this study, we use esophageal manometry to measure transpulmonary pressure between preemptive application of APRV and low tidal volume ventilation in our clinically applicable porcine extrapulmonary ARDS model. We demonstrate that the transpulmonary pressures are similar between the two ventilation strategies, despite the increased tidal volumes and plateau pressures in the APRV group, and that APRV was able to limit increases in chest wall elastance.

## Methods

All experiments were performed in accordance with National Institutes of Health guidelines in the use of laboratory animals and approved by the SUNY Upstate Medical University Institutional Animal Care and Use Committee (IACUC). The study was terminated upon achieving statistical significance between the two groups, according to the IACUC guidelines and study protocol. Female Yorkshire pigs (32–36 kg) were anesthetized using a continuous infusion of ketamine/xylazine to maintain a surgical plane of anesthesia. Animals were continuously monitored by the investigators for the duration of the experiment. Under sterile conditions, animals underwent tracheostomy and arterial and venous catheterization. The animals were connected to a Drӓger (Evita Infinity V500, Lübeck, Germany) ventilator and ventilated initially with (*V*_t_) 10 cc kg^−1^, positive end-expiratory pressure (PEEP) of 5 cmH_2_O, respiratory rate (RR) of 12 breaths min^−1^, and F_i_O_2_ 100 %.

A pulse index continuous cardiac output (PiCCO) catheter (Pulsion Medical Systems, Germany) was placed in the femoral artery with hourly injections to assess cardiac index and global end-diastolic index. A cystostomy was performed for continuous urine output and hourly IAP monitoring (ConvaTec Inc. NJ). Intra-abdominal hypertension was defined as sustained or repeated elevation in IAP (>12 mmHg) and abdominal compartment syndrome as repeated elevation in IAP (>20 mmHg) associated with new organ dysfunction according to consensus criteria [13]. The esophageal catheter was placed by first advancing the balloon into the stomach, where placement was confirmed by a transient increase in pressure during abdominal compression, then retracting it to the middle third of the esophagus with placement confirmed by noting cardiac oscillation and respiratory variation in the waveform [14]. Baseline (BL) measurements were taken after surgical preparation and prior to injury.

Extrapulmonary lung injury was induced using a previously established double-hit model of ischemia reperfusion and fecal peritonitis [11, 12, 15]. Briefly, the superior mesenteric artery was clamped for 30 min and released to induce intestinal ischemia. Peritoneal sepsis was induced by performing a cecotomy and mixing feces with blood to create a fecal clot, which was then implanted into the peritoneum. Time zero (T0) measurements were taken immediately after induction of the double-hit injury and upon closure of the abdomen. The animals were subsequently randomized into two groups: low tidal volume (LVt) ventilation or APRV.

LVt group (*n* = 3): Animals were transitioned from the baseline settings to low tidal volume settings of *V*_t_ 6 cc kg^−1^, PEEP of 5 cmH_2_O, RR 12 breaths min^−1^, and F_i_O_2_ 21 %. All ventilator adjustments were made in accordance with the ARDSnet guidelines with PEEP and F_i_O_2_ titrated according to SpO_2_ and P_a_O_2_ as outlined by the “Lower PEEP/higher F_i_O_2_ scale”. RR was titrated according to pH and P_a_CO_2_ and *V*_t_ was reduced to accommodate the plateau pressure (*P*_plat_) if values exceed 30 cmH_2_O.

APRV group (*n* = 3): APRV was applied and guided using a previously described protocol by Habashi [16]. Animals were ventilated at an inspiratory pressure *P*_High_ set at the *P*_plat_ established during the volume cycle setting used for BL measurements (16–22 cmH_2_O) for a time (*T*_High_) of 4.0–4.5 s, which was set to occupy approximately 90 % of the total ventilator cycle time. The release pressure (*P*_Low_) was set at 0 cmH_2_O to minimize expiratory resistance and maximize the peak expiratory flow rate. *P*_Low_ was applied for a time (*T*_Low_) to terminate the end-expiratory flow rate at 75 % of the peak expiratory flow rate, which was between 0.32 and 0.37 s. *P*_High_, *T*_High_, *T*_Low_, and F_i_O_2_ were titrated throughout the study according to pulmonary parameters, P_a_O_2_ and P_a_CO_2_.

### Resuscitative protocol

Antibiotics, fluid, and vasopressor administration were guided by the Surviving Sepsis campaign [17]. Broad-spectrum antibiotics (vancomycin 1 g and piperacillin/tazobactam 3.375 g) were administered following abdominal closure and throughout the study every 12 and 8 h, respectively. Animals were provided with continuous maintenance intravenous fluid resuscitation and boluses as needed with Lactated Ringers to maintain a mean arterial pressure (MAP) >65 mmHg. Continuous infusion of norepinephrine was initiated when the animal was no longer fluid responsive, followed by vasopressin and epinephrine. Rocuronium was initiated if spontaneous respiratory effort was demonstrated in order to standardize animals across groups.

### Physiologic measurements

Hemodynamics were monitored continuously (Intellivue MP-90, Phillips Healthcare, Irvine, CA) using Edwards transducers (Pressure Monitoring Kit, Edwards Lifesciences, Irvine, CA). Blood gases were measured every 1–3 h with a Roche blood gas analyzer (Cobas b221, Basel, Switzerland).

### Pulmonary parameters

Pulmonary parameters were measured or calculated by the Drӓger ventilator. The end-expiratory pressure in APRV was taken to be the lowest value during the expiratory release phase after accounting for tracheal tube compensation. The respiratory system may be partitioned into the lung and the chest wall, and the plateau pressure (*P*_plat_) distributed across the respiratory system may also be divided into the corresponding transpulmonary pressure (*P*_l_) and pleural pressure (*P*_pl_) [18, 19].

1 $$ {P}_{\mathrm{pl}\mathrm{at}} = {P}_{\mathrm{l}} + {P}_{\mathrm{pl}} $$

Similarly, the sum of the lung (*E*_l_) and chest wall (*E*_cw_) elastance represents the elastance of the entire respiratory system (*E*_rs_) (Eq. 2) [18].

2 $$ {E}_{\mathrm{rs}} = {E}_{\mathrm{cw}} + {E}_{\mathrm{l}} $$

The elastance calculated by the ventilator (standardly reported as compliance on the monitor) represents the elastance of the respiratory system (Eq. 3) but does not distinguish lung from chest wall elastance.

3 $$ {E}_{\mathrm{rs}} = \left({P}_{\mathrm{plat}}\hbox{--}\ \mathrm{PEEP}\right)/{V}_{\mathrm{t}} $$

The use of esophageal manometry to determine the partitioning of respiratory system elastance into chest wall and lung elastance was first described in a thesis by Buytendijk in 1949 [20, 21] although several methods of direct [22, 23] and indirect [14, 24, 25] measurements have since been described. The change in *P*_es_ between inspiration and expiration (Δ*P*_Es_) approximates the change in *P*_pl_ [26, 27]; thus, the elastance of the chest wall may be calculated as follows [28]:

4 $$ \Delta {P}_{\mathrm{Es}}/{V}_{\mathrm{t}} = {E}_{\mathrm{cw}} $$

Therefore, the distribution of *P*_aw_ to the lung (*P*_l_) and chest wall (*P*_pl_) can be calculated based on the ratios of lung elastance and chest wall elastance to the respiratory system elastance, respectively (Eqs. 5 and 6) [9, 18].

5 $$ {P}_{\mathrm{pl}} = {P}_{\mathrm{pl}\mathrm{at}} \cdot \left({E}_{\mathrm{cw}}/{E}_{\mathrm{rs}}\right) $$

6 $$ {P}_{\mathrm{l}} = {P}_{\mathrm{plat}} \cdot \left({E}_{\mathrm{l}}/{E}_{\mathrm{rs}}\right) $$

### Necropsy

After 48 h, the experimental protocol was terminated. Animals were euthanized with Fatal-Plus (1 mL 10 lbs^−1^ intravenous), cardiac death confirmed, and necropsy performed. The lungs were removed and inflated to 25 cmH_2_O, using stepwise increases in PEEP to standardize lung volume history, and grossly photographed. The left lung was filled with 10 % formalin to a height of 25 cmH_2_O, clamped and submerged in formalin. The right middle lobe was lavaged with 60 mL of normal saline to collect bronchoalveolar lavage fluid (BALF). The concentrations of interleukin-6 and -8 (IL-6 and IL-8) were determined using enzyme-linked immunosorbent assay (ELISA) quantification according to manufacturer’s recommendations. Western blot analyses of surfactant protein A (SP-A) and B (SP-B) abundance as well as determination of total protein were performed as described previously [11].

### Quantitative histology

The quantitative histological assessment of the lung was based on image analysis of 120 photomicrographs (10 per animal) made at high-dry magnification following a validated, blinded, systematic sampling protocol [15]. Each photomicrograph was scored using a 4-point scale for each of the five parameters: atelectasis, fibrinous deposits and blood in air space, vessel congestion, alveolar wall thickness, and leukocytes.

### Statistics

The study was terminated upon achieving statistical significance between the two groups, according to the IACUC guidelines to reduce the number of animals used for experimentation and the study protocol. Data are reported as mean ± SEM. Repeated measures ANOVA was used to compare differences within and between treatment groups for continuous parameters and post hoc Tukey’s tests if significance was found in the group*time effect. Categorical data were compared using an unpaired Student’s *t* test. Quantitative histological assessment was analyzed using Mann-Whitney *U* test after testing for normality. *p* values <0.05 were considered significant. Analyses were performed using JMP (version 10, Cary, NC).

## Results

### Hemodynamics

Both LVt and APRV pigs had a precipitous decline in MAP in the hour following injury with a steady decline thereafter (Table 1). The MAP was similar in both groups and was maintained above 65 mmHg with fluid and vasopressor support (*p* > 0.05). The total volume of fluid infused over the course of the experiment was similar between LVt (36.0 ± 7.5 L) and APRV (47.8 ± 7.2 L; *p* > 0.05; Table 1). The cardiac index (LVt 3.3 ± 0.7 L min^−1^ m^−2^; APRV 2.0 ± 0.3 L min^−1^ m^−2^) and global end-diastolic index (LVt 563.7 ± 167.8 mL · min^−2^; APRV 314.0 ± 111.9 mL · min^−2^), as measured by the PiCCO catheter, were similar between groups (*p* > 0.05; Table 1).

**Table 1:** Hemodynamic data and organ injury in low tidal volume (LVt) versus airway pressure release ventilation (APRV)

		Baseline	12 h	24 h	36 h	48 h	*p* value
MAP	LVt	123.7 ± 15.8	73.3 ± 3.3	77.7 ± 2.7	76.3 ± 4.3	71.3 ± 3.3	0.0811
(mmHg)	APRV	109.3 ± 5.2	86.7 ± 3.9	69.0 ± 1.2	73.0 ± 1.2	72.7 ± 1.5	
Cardiac index	LVt	3.2 ± 0.6	2.6 ± 0.3	2.4 ± 0.2	3.7 ± 0.4	3.3 ± 0.7	0.8551
(L min^−1^ m^−2^)	APRV	2.8 ± 0.5	2.0 ± 0.1	2.1 ± 0.1	2.5 ± 0.3	2.0 ± 0.3	
Global end-diastolic index	LVt	596.0 ± 78.0	528.0 ± 139.9	467.7 ± 135.3	543.0 ± 145.0	563.7 ± 167.8	0.0604
(mL min^−2^)	APRV	475.7 ± 141.9	484.7 ± 146.5	407.0 ± 129.0	391.3 ± 116.4	314.0 ± 111.9	
Intra-abdominal pressure	LVt	2.3 ± 1.6	13.6 ± 2.4	11.8 ± 2.0	13.1 ± 4.5	11.3 ± 5.0	<0.0001
(cmH_2_O)	APRV	0.9 ± 0.5	15.0 ± 2.1	19.5 ± 2.4	25.8 ± 8.5	21.8 ± 0.8	
Cumulative urine output (L)	LVt	0.8 ± 0.7	3.8 ± 0.9	6.2 ± 1.2	9.1 ± 1.2	16.7 ± 5.0	0.599
	APRV	0.5 ± 0.2	2.4 ± 0.4	4.6 ± 0.7	7.3 ± 0.8	9.1 ± 1.1	
Urine output (mL kg^−1^)	LVt	22.2 ± 18.2	6.4 ± 2.0	5.0 ± 1.5	6.3 ± 1.1	12.2 ± 6.9	0.5469
	APRV	14.4 ± 4.0	4.0 ± 1.4	6.8 ± 1.3	4.9 ± 0.6	2.5 ± 0.5	
Cumulative fluids administered (L)	LVt	2.1 ± 0.3	11.9 ± 1.7	19.2 ± 2.9	25.7 ± 3.9	36.0 ± 7.5	0.3266
	APRV	1.5 ± 0.1	14.3 ± 0.5	22.9 ± 0.5	32.5 ± 0.7	47.8 ± 7.2	
Blood urea nitrogen	LVt	5.0 ± 0.4	7.3 ± 1.2	8.7 ± 2.4	9.1 ± 2.7	7.7 ± 1.9	0.9543
(mg dL^−1^)	APRV	5.7 ± 0.5	7.0 ± 0.5	9.2 ± 0.8	11.8 ± 1.2	11.1 ± 2.8	

Only intra-abdominal pressure was significant between groups over time; however, there was no significant difference at any individual time point

### Pulmonary data

The end-expiratory release pressure was significantly greater in APRV as compared with LVt (*p* < 0.05; Table 2), despite a *P*_Low_ of 0 cmH_2_O, demonstrating the importance of setting the *T*_Low_ appropriately to ensure the end-expiratory pressure never has the time to actually reach 0 cmH_2_O. Consistent with previous studies [11, 29], the tidal volumes in the APRV group (13.3. ± 0.6 cc kg^−1^) were significantly greater than those in the LVt group (5.6 ± 0.3 cc kg^−1^; *p* < 0.05; Table 2). In one LVt animal, the *P*_plat_ became greater than 30 cmH_2_O at T43 (although the corresponding transpulmonary pressures was 23.9 cmH_2_O) and the *V*_t_ was decreased to maintain *P*_plat_ below 30 cmH_2_O as per the ARDSnet protocol [3]; however, this led to prompt desaturation (SpO_2_ <88 %) that ultimately required titrating PEEP and F_i_O_2_ upward (requiring an F_i_O_2_ of 50 % and a PEEP of 10 cmH_2_O to maintain adequate oxygen saturation by T48). By the study end, LVt animals had significantly greater F_i_O_2_ requirements (37.7 ± 6.7 %) as compared with the APRV pigs, all of which were maintained on an F_i_O_2_ of 21 % throughout the study (21.0 ± 0.0 %; *p* < 0.05; Table 2). Significant differences in the P_a_O_2_/F_i_O_2_ ratio between APRV and LVt were revealed by T30 and persisted until T48 with final P_a_O_2_/F_i_O_2_ ratio of 226.2 ± 17.1 in LVt and 465.7 ± 66.5 in APRV (*p* < 0.05) with all animals in the LVt group meeting the Berlin criteria for mild ARDS [30] by T36 (Table 2).

**Table 2:** Pulmonary data in low tidal volume (LVt) versus airway pressure release ventilation (APRV)

		Baseline	12 h	24 h	36 h	48 h	*p* value
Plateau pressure (cmH_2_O)	LVt	16.7 ± 2.7	14.5 ± 0.5	18.2 ± 1.1	22.0 ± 1.9	22.2 ± 2.0	0.0188§
	APRV	18.2 ± 2.2	22.8 ± 2.5*	27.0 ± 0.6*	31.0 ± 0.6*	34.3 ± 0.9*	
Transpulmonary pressure (cmH_2_O)	LVt	12.7 ± 3.8	9.6 ± 1.7	11.2 ± 0.1	13.8 ± 1.7	14.9 ± 1.9	0.1116
	APRV	12.7 ± 3.2	12.6 ± 1.3	16.4 ± 1.5	14.6 ± 2.1	17.3 ± 2.9	
Pleural pressure (cmH_2_O)	LVt	3.9 ± 1.3	4.9 ± 1.4	6.9 ± 1.1	8.2 ± 0.3	7.6 ± 0.5	<0.0001§
	APRV	5.5 ± 1.0	10.2 ± 1.2*	10.6 ± 1.2	16.1 ± 1.4*	17.4 ± 3.5*	
End-expiratory pressure (set) (cmH_2_O)	LVt	5.0 ± 0.0	5.0 ± 0.0	5.0 ± 0.0	6.0 ± 1.0	6.6 ± 1.7	<0.0001§
	APRV	0.0 ± 0.0*	0.0 ± 0.0*	0.0 ± 0.0*	0.0 ± 0.0*	0.0 ± 0.0*	
End-expiratory pressure (measured) (cmH_2_O)	LVt	5.1 ± 0.1	5.0 ± 0.1	5.2 ± 0.1	6.2 ± 1.2	6.6 ± 1.7	<0.0001§
	APRV	5.1 ± 0.1	10.7 ± 0.9*	11.8 ± 0.6*	14.2 ± 1.1*	17.8 ± 2.0*	
Tidal volume (cc kg^−1^)	LVt	10.2 ± 0.1	6.0 ± 0.0	6.0 ± 0.0	5.9 ± 0.0	5.6 ± 0.3	<0.0001§
	APRV	10.0 ± 0.1	9.7 ± 0.3*	11.0 ± 0.8*	12.3 ± 0.6*	13.3 0.6*	
Respiratory system elastance (cmH_2_O L^−1^)	LVt	31.2 ± 7.9	43.5 ± 4.2	59.8 ± 6.3	72.8 ± 8.3	76.4 ± 9.4	<0.0001§
	APRV	35.8 ± 6.0	34.2 ± 4.8	38.5 ± 5.5	38.0 ± 5.6*	33.4 ± 4.6*	
Lung elastance (cmH_2_O L^−1^)	LVt	24.4 ± 9.4	29.3 ± 6.7	36.7 ± 1.6	45.7 ± 6.3	50.3 ± 8.3	<0.0001§
	APRV	29.7 ± 6.6	18.8 ± 2.6	23.8 ± 5.1	18.8 ± 4.8*	17.6 ± 5.1*	
Chest wall elastance (cmH_2_O L^−1^)	LVt	6.8 ± 1.9	14.2 ± 3.9	23.1 ± 4.8	27.0 ± 2.6	26.2 ± 2.9	0.0377§
	APRV	10.6 ± 1.8	15.4 ± 2.3	14.7 ± 1.0	19.2 ± 1.1*	15.8 ± 0.5*	
FiO_2_ (%)	LVt	1.00 ± 0.00	0.24 ± 0.03	0.27 ± 0.05	0.33 ± 0.03	0.37 ± 0.07	<0.0001§
	APRV	1.00 ± 0.00	0.21 ± 0.00	0.21 ± 0.00	0.21 ± 0.00*	0.21 ± 0.00	
P_a_O_2_/FiO_2_ (mmHg)	LVt	550.4 ± 20.1	429.4 ± 36.6	411.3 ± 39.2	269.6 ± 6.4	226.2 ± 17.1	<0.0001§
	APRV	556.8 ± 27.4	443.5 ± 29.4	416.8 ± 26.5	374.6 ± 13.8*	465.7 ± 66.5*	

The end-expiratory pressure set on the ventilator with LVt (PEEP) and APRV (*P*_Low_) are distinguished from the actual end-expiratory pressure measured at the level of the trachea. *p* value (right column) following RM ANOVA with ^§^ *p* < 0.05 considered significant. *p < 0.05 LVt versus APRV following post hoc analysis with Tukey’s test

**Fig. 1:**
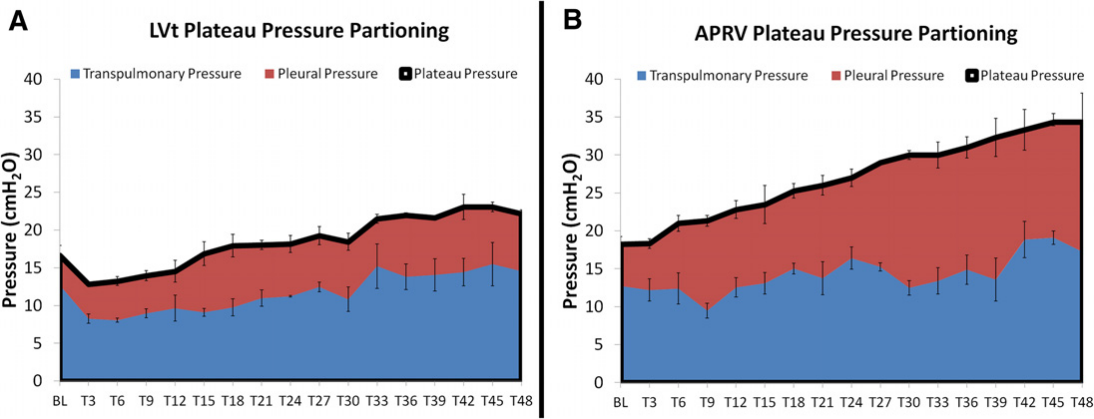
The plateau pressure (*black line* at top of the red area curve) in LVt (**a**) is significantly lower than that of APRV (**b**) yet the transpulmonary pressures (*blue*) are statistically similar between groups. This demonstrates that the increases in plateau pressure in APRV reflects and increase in pleural pressure (*red*)

The plateau pressures in both groups increased steadily over the course of the study with a significantly lower plateau pressure in LVt (22.2 ± 2.0 cmH_2_O) as compared with APRV (34.3 ± 0.9 cmH_2_O; *p* < 0.05; Table 2). Over time, the *P*_pl_ in APRV increased from 5.5 ± 1.0 cmH_2_O at T0 to 17.4 ± 3.5 cmH_2_O at T48 whereas the *P*_pl_ in LVt remained relatively stable from 4.0 ± 1.3 to 7.6 ± 0.5 cmH_2_O leading to a significant difference between the two groups by T48 (*p* < 0.05; Fig. 1). The *P*_l_ in the LVt group (14.6 ± 2.1cmH_2_O) was similar to that of the APRV group (17.3 ± 2.9 cmH_2_O; *p* > 0.05). In combination, these data suggest that the majority of the increased *P*_plat_ in APRV was being distributed as *P*_pl_, directed towards the chest wall, rather than increasing lung stress. Finally, the elastance of the lung, chest wall, and respiratory system were all significantly greater in LVt as compared with APRV (all *p* < 0.05; Table 2). By the end of the 48-h study, the lung elastance increased by 154 ± 78 % in the LVt group but was reduced by 43.2 ± 5.2 % in the APRV group.

### Organ injury

Animals in the APRV group had an increase in IAP (21.8 ± 0.8 cmH_2_O) as compared with LVt (11.3 ± 5.0 cmH_2_O; *p* < 0.05; Table 1), consistent with the increase in *P*_pl_ seen in APRV. Despite the measured increased IAP in APRV, there was no clinical evidence of reduced end-organ perfusion in either group and no animal required a decompressive laparotomy for abdominal compartment syndrome. Both groups had similar blood urea nitrogen levels, and the total urine output between the two groups was similar (*p* > 0.05; Table 1).

### Gross pathology and quantitative histology

**Fig. 2:**
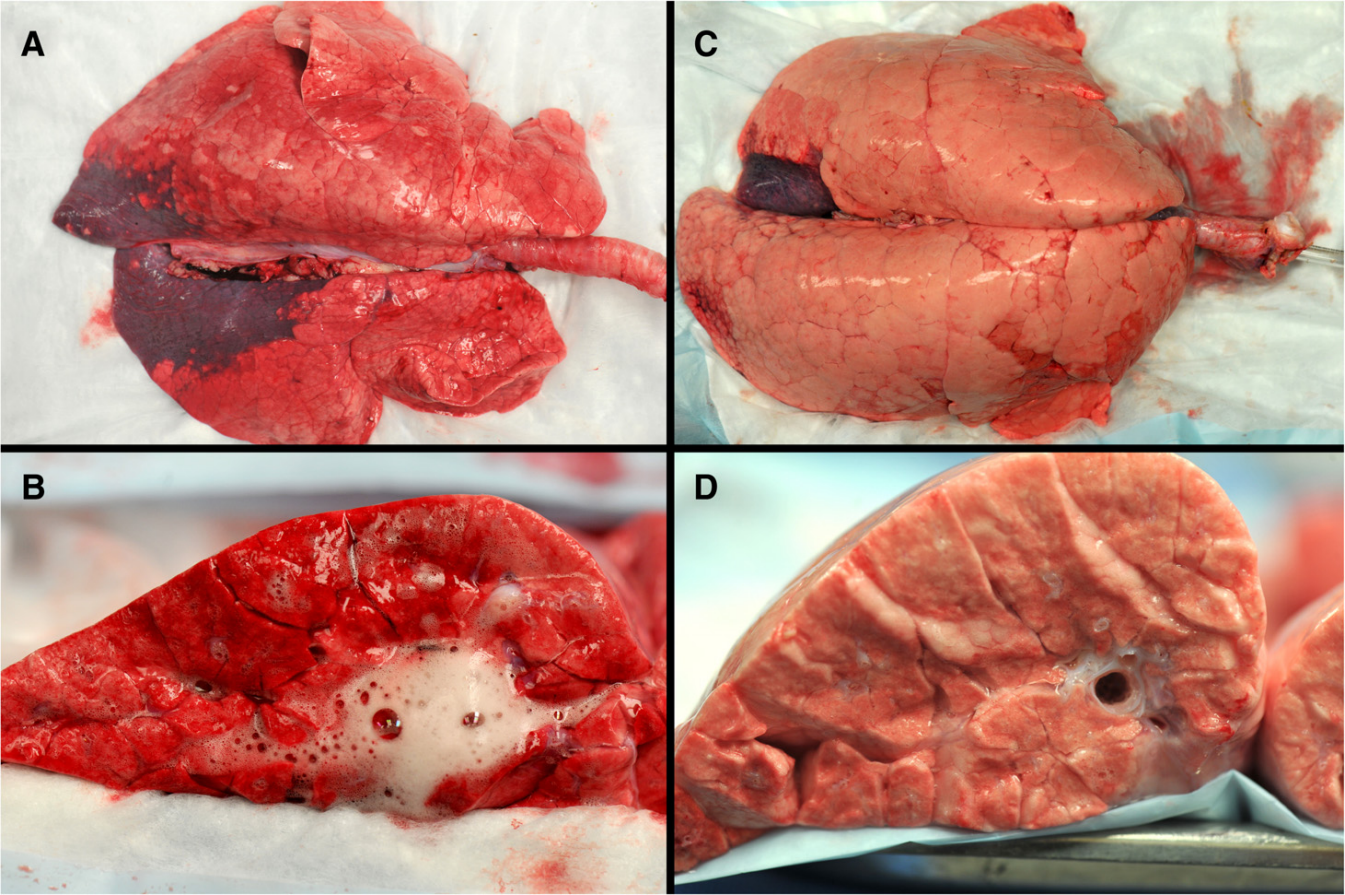
Low tidal volume ventilation lungs (**a**) inflated heterogeneously with prominent dependent and basilar atelectasis and the cut surface (**b**) revealing airway edema. Airway Pressure Release Ventilation gross lungs (**c**) and cut surface (**d**) were pink, light and inflated homogeneously without airway edema

The lungs of the LVt group inflated heterogeneously with predominant basilar and dependent atelectasis; the majority of which could be recruited with persistent pressure (Fig. 2a). The cut surfaces of the LVt group were erythematous and had both interlobular septal edema and bronchial edema (Fig. 2b). The lungs of the APRV group were pink, light, and inflated homogeneously (Fig. 2c), and the cut surface of the lung demonstrated interlobular septal edema but little bronchial edema (Fig. 2d). Two of the three pigs in each group demonstrated small bowel dilatation consistent with ileus as well as bowel wall edema. All of the pigs in both groups demonstrated gastric ulceration ranging from hyperemia to gross hemorrhagic ulcers. The wet-dry weight for the LVt group (7.0 ± 0.3) was similar to the APRV group (7.6 ± 0.9; *p* > 0.05).

**Fig. 3:**
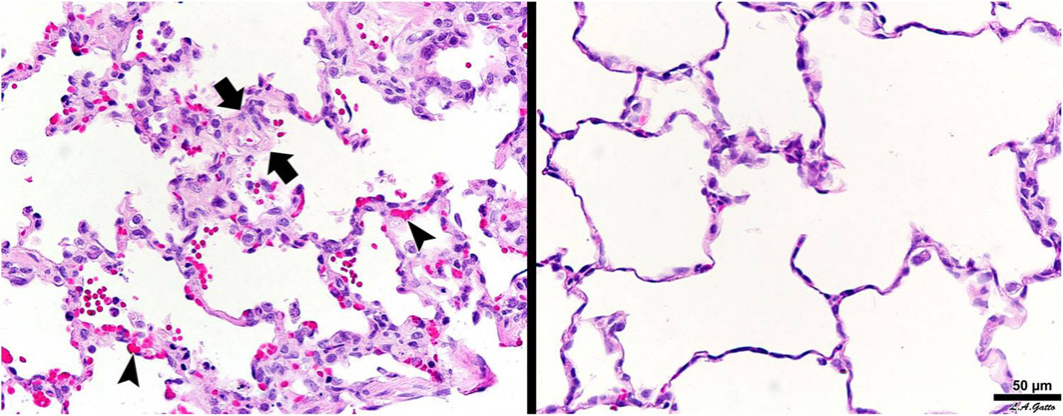
Low tidal volume (LVt; *left*) demonstrated increased alveolar wall thickness (*between arrows*) and vessel congestion (*arrowheads*) as compared with Airway Pressure Release Ventilation (APRV; *right*)

The lungs in the LVt group demonstrated a significant increase in alveolar wall thickening as compared with APRV (*p* < 0.05; Fig. 3). There was also a significant increase in intra-alveolar hemorrhage in the LVt group as compared with APRV (*p* < 0.05), with the luminal erythrocytes noted to be intact with no signs of hemolysis. Although not statistically significant, LVt had a relative increase in vessel congestion (Fig. 3), atelectasis, and fibrinous deposits (*p* > 0.05).

### Bronchoalveolar lavage

There was a trend towards an increase in total protein in the BALF in the LVt group (1271.5 ± 590.6 μg mL^−1^) as compared with APRV (300.9 ± 9.8 μg mL^−1^) although this did not reach statistical significance (*p* = 0.18). SP-A in the BALF was significantly reduced in the LVt group as compared with APRV (*p* < 0.05) although SP-B was not significantly different between the two groups (*p* = 0.09). The BALF concentrations of IL-8 were similar between LVt (145.8 ± 115.5 pg mL^−1^) and APRV (79.4 ± 56.6 pg mL^−1^; *p* > 0.05). The BALF concentrations of IL-6 was relatively greater in the LVt group (2677 ± 1060 pg mL^−1^) as compared with APRV (342.1 ± 146.9 pg mL^−1^; *p* > 0.05).

## Discussion

The transpulmonary pressure between APRV and LVt, however, was similar suggesting that the *P*_plat_ and *V*_t_ in APRV did not lead to increased lung stress. This is in spite of the fact that the APRV group was associated with an elevated *P*_plat_ and greater *V*_t_, as compared with LVt, both of which are currently considered injurious [3]. Thus, the majority of the plateau pressure in the APRV group was being distributed as pleural pressure, applied to the chest wall, an important finding in this extrapulmonary lung injury model associated with increased chest wall elastance. In addition, APRV preserved surfactant protein-A concentrations and reduced epithelial permeability, as measured by BALF protein. APRV also preserved lung elastance and reduced lung injury by histopathologic scoring.

### Intra-abdominal pressure and chest wall elastance

ARDS can be largely subdivided by its original etiology: pulmonary (i.e., pneumonia and aspiration) and extrapulmonary (i.e., sepsis, hemorrhage, peritonitis, systemic inflammatory response syndrome, and multi-organ trauma). Although the two ARDS phenotypes may have similar pathophysiologic outcomes, the pathogenesis and treatment are different [1]. Whereas increased lung and respiratory system elastance is found in both pulmonary and extrapulmonary ARDS, an increase in chest wall elastance is primarily associated with extrapulmonary ARDS [1]. The chest wall consists of the anterior and posterior thoracic cage and the diaphragm, which serves as a pliable separation between the abdominal and thoracic cavities [31]. Approximately half of the IAP is transmitted to the intrathoracic space [31, 32]; thus, increased IAP is one of the most common causes of increased chest wall elastance in extrapulmonary ARDS and has been associated with increased pulmonary edema, atelectasis, and lung neutrophil activation [19]. Body wall edema can further increase elastance of both the chest wall and abdomen [33].

Intra-abdominal hypertension has been observed in 54.4 % of medical and 65.0 % of surgically critically ill patients [18]. The IAP at end-inspiration has been shown to be approximately four times greater in the surgical ARDS groups as compared with the medical ARDS group [34]. An increase in IAP transmits stress to the thoracic cavity causing a decrease in functional residual capacity, ventilation-perfusion mismatching, a shift in the volume-pressure curve of the chest to the right, and compression atelectasis [31, 32, 34, 35]. The negative effects of increased IAP on the thoracic cavity can be attenuated by increasing the pleural pressure, effectually placing an opposing force on the IAP [36, 37]. Since pleural pressure and IAP have a linear relationship [1], the combination of a prolonged inspiratory time and increased pleural pressure in the APRV group further increased the measured IAP [38] and is likely the mechanism of decreased chest wall elastance in this group.

Despite the increase in IAP, APRV improved lung elastance while maintaining a physiologic P_a_O_2_/F_i_O_2_ ratio. The importance of delivering a pressure sufficient to shift the volume-pressure curve of the chest back to the left and increase diaphragm tension at end-expiration to prevent the negative effects of IAP transmission to the chest wall was established in a study of increasing PEEP [39]. In the current study, the extended time at the *P*_plat_ in the APRV group similarly opposed the effects of increased IAP. Conversely, the lungs of the LVt pigs universally had bibasilar atelectasis suggesting that the PEEP scale guided by the ARDSnet protocol was insufficient to oppose the force of the IAP on the lower lung lobes. This is supported by the significant increase in lung elastance in the LVt group by 48 h, and the trend suggested that the lung elastance would have increased further had the animals not reached the termination point of the study.

### Plateau pressure versus pleural pressure

As the IAP increases and the chest wall becomes stiffer, more of the *P*_plat_ is generated as *P*_pl_ rather than *P*_l_ [19, 33, 40]. In patients with high *E*_cw_, limiting *P*_plat_ could worsen oxygenation and enable lung derecruitment if the transpulmonary pressure is not considered, whereas an appropriately high plateau pressure could improve oxygenation and lung/chest wall elastance [40]. In a porcine model by Kubiak et al. [33], pneumoperitoneum was established and, as IAP increased, *P*_plat_ and *P*_pl_ increased but *P*_l_ did not, whereas ventilating to similar *P*_plat_ in a desufflated abdomen led to a significant increase in *P*_l_. Thus, that study demonstrates that in a patient population with a compromised chest wall, *P*_plat_ is a poor surrogate for *P*_l_, and setting the upper limit for *P*_aw_ at 30 cmH_2_O may not be realistic without considering the underlying chest wall mechanics [33].

In patients with influenza A (H1N1)-induced ARDS referred for extracorporeal membrane oxygenation (ECMO), Grasso et al. [41] determined that there was a subset of patients in whom the majority of pressure applied by the ventilator was being transmitted to the stiff chest wall rather than to recruiting the lung [41]. The authors determined that targeting end-inspiratory *P*_l_ rather than the respiratory system *P*_plat_ significantly improved patient oxygenation such that 50 % of patients that previously met ECMO criteria no longer did [41]. In this current study, the LVt pig that achieved a *P*_plat_ greater than 30 cmH_2_O had a corresponding *P*_l_ of 23.4 cmH_2_O and dropping the tidal volumes to accommodate the *P*_plat_ led to prompt desaturation.

It has previously been demonstrated in an in vivo study that APRV, with an extended time at the *P*_plat_, improves alveolar recruitment and alveolar surface area, suggesting that these larger tidal volumes are being distributed over a greater number of open alveoli, reducing the dynamic strain on individual alveoli [42]. In a prospective study by Chiumello et al. [43], comparing control patients with those with acute lung injury or ARDS, increasing PEEP from 5 to 15 cmH_2_O led to a decrease in lung, chest wall, and respiratory system elastance. Additional time at a greater pressure (PEEP or plateau pressure) therefore allows for increased alveolar recruitment and distribution of the tidal volume over a larger surface area of alveoli, reducing overall lung stress. Protective mechanical ventilation should be instituted early and consideration given towards increasing PEEP or extending time at the *P*_plat_ to optimize recruitment while limiting the potential negative effects of larger *V*_t_.

## Conclusions

In this study, we have demonstrated that the early application of APRV improves oxygenation and maintains surfactant as compared with LVt applied immediately following injury. APRV had greater plateau pressures and tidal volumes as compared with LVt yet the transpulmonary pressures between the groups were similar. Thus, APRV represents a safe and effective ventilation mode in patients at risk for the development of extrapulmonary lung injury.

## Abbreviations

ARDS: acute respiratory distress syndrome
APRV: airway pressure release ventilation
LV_t_: low tidal volume ventilation
*E*_cw_: chest wall elastance
*E*_rs_: respiratory system elastance
*E*_l_: lung elastance
IACUC: Institutional Animal Care and Use Committee
*V*_t_: tidal volume
PEEP: positive end-expiratory pressure
RR: respiratory rate
IAP: intra-abdominal pressure
BL: baseline
T0: time zero
*P*_plat_: plateau pressure
*P*_l_: transpulmonary pressure
*P*_pl_: pleural pressure
∆*P*_es_: change in esophageal pressure
BALF: bronchoalveolar lavage fluid
SP-A: surfactant protein A
SP-B: surfactant protein B
